# Influence of the freezing level on atmospheric rivers in High Mountain Asia: WRF case studies of orographic precipitation extremes

**DOI:** 10.1007/s00382-023-06929-x

**Published:** 2023-08-28

**Authors:** Deanna Nash, Leila M. V. Carvalho, Jonathan J. Rutz, Charles Jones

**Affiliations:** 1 Center for Western Weather and Water Extremes, Scripps Institution of Oceanography, University of California, San Diego, CA 92093 USA; 2 Department of Geography and Earth Research Institute, University of California, Santa Barbara, CA 93106 USA

**Keywords:** Atmospheric rivers, High Mountain Asia, Orographic precipitation, Freezing level

## Abstract

Atmospheric rivers (ARs) reach High Mountain Asia (HMA) about 10 days per month during the winter and spring, resulting in about 20 mm day^-1^ of precipitation. However, a few events may exceed 100 mm day^-1^, providing most of the total winter precipitation and increasing the risk of precipitation-triggered landslides and flooding, particularly when the height of the height of the 0 ^∘^C isotherm, or freezing level is above-average. This study shows that from 1979 to 2015, integrated water vapor transport (IVT) during ARs that reach Western HMA has increased 16% while the freezing level has increased up to 35 m. HMA ARs that have an above-average freezing level result in 10–40% less frozen precipitation compared to ARs with a below-average freezing level. To evaluate the importance of these trends in the characteristics of ARs, we investigate mesoscale processes leading to orographic precipitation using Advanced Weather Research and Forecasting (ARW-WRF) simulations at 6.7 km spatial resolution. We contrast two above- and below- average freezing level AR events with otherwise broadly similar characteristics and show that with a 50–600 m increase in freezing level, the above-average AR resulted in 10–70% less frozen precipitation than the below-average event. This study contributes to a better understanding of climate change-related impacts within HMA’s hydrological cycle and the associated hazards to vulnerable communities living in the region.

## Introduction

In High Mountain Asia (HMA), cool-season precipitation and the resulting spring and summer glacial melt provides water resources for hundreds of millions of people, but also presents risks for many extreme weather conditions (Kääb et al. 2012; Hewitt 2005). Recent work has shown that atmospheric rivers (ARs), long conduits of strong moisture transport, are significant contributors to winter and spring precipitation in HMA (Nash et al. 2021). ARs occur in a variety of locations across the globe and are associated with extreme precipitation, flooding, lightning, landslides and anomalous snow accumulation (Cannon et al. 2018; Nash and Carvalho 2020; Oakley et al. 2018; Zhu and Newell 1994, among others). In HMA, ARs contribute to extreme precipitation and are associated with flood events in the Nepal and Bay of Bengal areas (Thapa et al. 2018; Yang et al. 2017). Nash et al. (2021) found and characterized three distinct types of ARs producing above-average precipitation in northwestern, western, and eastern HMA. Moreover, they determined that there are typically between 9 and 11 HMA ARs per month in the winter and spring, contributing between 40 and 60% of total seasonal precipitation. However, on some occasions, a single strong AR event can provide up to a quarter of that precipitation, with precipitation totals exceeding 100 mm day^-1^ increasing rainfall-related risks, such as landslides and flooding.

Many studies have investigated long-term climate trends over HMA. In western HMA, Norris et al. (2019) identified positive trends of cloud ice and liquid cloud, indicating the higher frequency of extratropical cyclones in recent years. Nash et al. (2021) demonstrated that of the three types of HMA ARs, Northwestern and Western HMA ARs are primarily associated with extratropical cyclones, where the warm, moist air from the AR is advected in the area ahead of the cold front. Given this information, it is likely there have been changes in the frequency or intensity of HMA ARs, although this has yet to be quantified. Furthermore, Wang et al. (2014) observed upward trends in the height of the 0 ^∘^C isotherm (hereafter, the freezing level) during summer in HMA. Changes in winter freezing levels have yet to be quantified in HMA, but increases in the freezing level are likely to result in decreased frozen precipitation, particularly during ARs. Previous studies have observed the increase of the freezing level during an AR, as extratropical cyclones associated with an AR are typically warmer than those without (Lundquist et al. 2008; Neiman et al. 2008, 2011). Above-average freezing levels during ARs can increase the likelihood of precipitation-related hazards because the fraction of rain to snow at higher elevations results in increased runoff and snow melt (Guan et al. 2016).

Espinoza et al. (2018) demonstrated that under the RCP 8.5 warming scenario, the frequency of HMA ARs is expected to increase by 6–8% while the intensity of integrated water vapor transport (IVT) is expected to remain the same between 2073 and 2096. Kirschbaum et al. (2020) showed that increases in extreme precipitation in HMA has the potential to increase landslides by 10–70% more in the years 2061–2100. Increases in ARs and their intensity could potentially increase precipitation and precipitation-related hazards; therefore, it is important to understand recent changes in AR properties to determine their influence on local warming and precipitation trends.

This study highlights the importance of long-term trends in the freezing level associated with HMA ARs by contrasting two events that both resulted in extreme precipitation across western HMA. These two events featured greatly differing freezing level heights and thus outcomes regarding precipitation-related hazards. Advanced Weather Research and Forecasting (ARW-WRF, hereafter WRF) simulations at 6.7 km resolution are used to differentiate between the mesoscale characteristics of these two events. The finer spatial resolution of this model largely overcomes the typical limitations of scarce observational data and coarse reanalysis resolution (> 27 km) amidst the complex topography of HMA. Focus is placed on mesoscale characteristics that are important to extreme precipitation, such as water vapor flux, the orientation of the AR relative to topography, the height of the freezing level, and the orographic mechanisms related to precipitation in the foothills of HMA.

The organization of this paper is as follows: Sects. 2 and 3 describes the data used for this analysis and outlines WRF model set up. Section 4.1 describes thermodynamic trends during HMA AR events using 36 years of dynamically downscaled reanalyses over HMA. We evaluate changes in the freezing level and moisture, focusing on areas where HMA ARs typically result in above-average precipitation during the winter. Sections 4.2 and 4.3 outlines the selection of two extreme AR events that had similar overall characteristics but had different freezing levels and precipitation amounts. Section 4.4 compares the synoptic patterns of both events. Using the WRF model, Sect. 4.5 examines the mesoscale meteorology of two ARs associated with extreme precipitation, emphasizing the differences between an AR event with an above- and below-average freezing level. We summarize our results in Sect. 5.

## Data

### AR detection: tARget v3

To detect ARs, we use the Tracking Atmospheric Rivers Globally as Elongated Targets (tARget) algorithm version 3 which was applied to global, 6-hourly ERA-Interim data from 1979 to 2015 (Guan and Waliser 2019). This AR detection algorithm is useful for the HMA region as it detects ARs via relative IVT intensity thresholds, which is particularly useful during the winter in HMA, as there is, on average, little to no moisture (Nash et al. 2021). Nash et al. (2021) identified three main types of ARs that reach HMA in winter and spring months using tARget v3. We use the resulting classification of HMA AR types in this study to focus on Northwestern and Western HMA AR Types that resulted in extreme precipitation.

### WRF setup

**Fig. 1:**
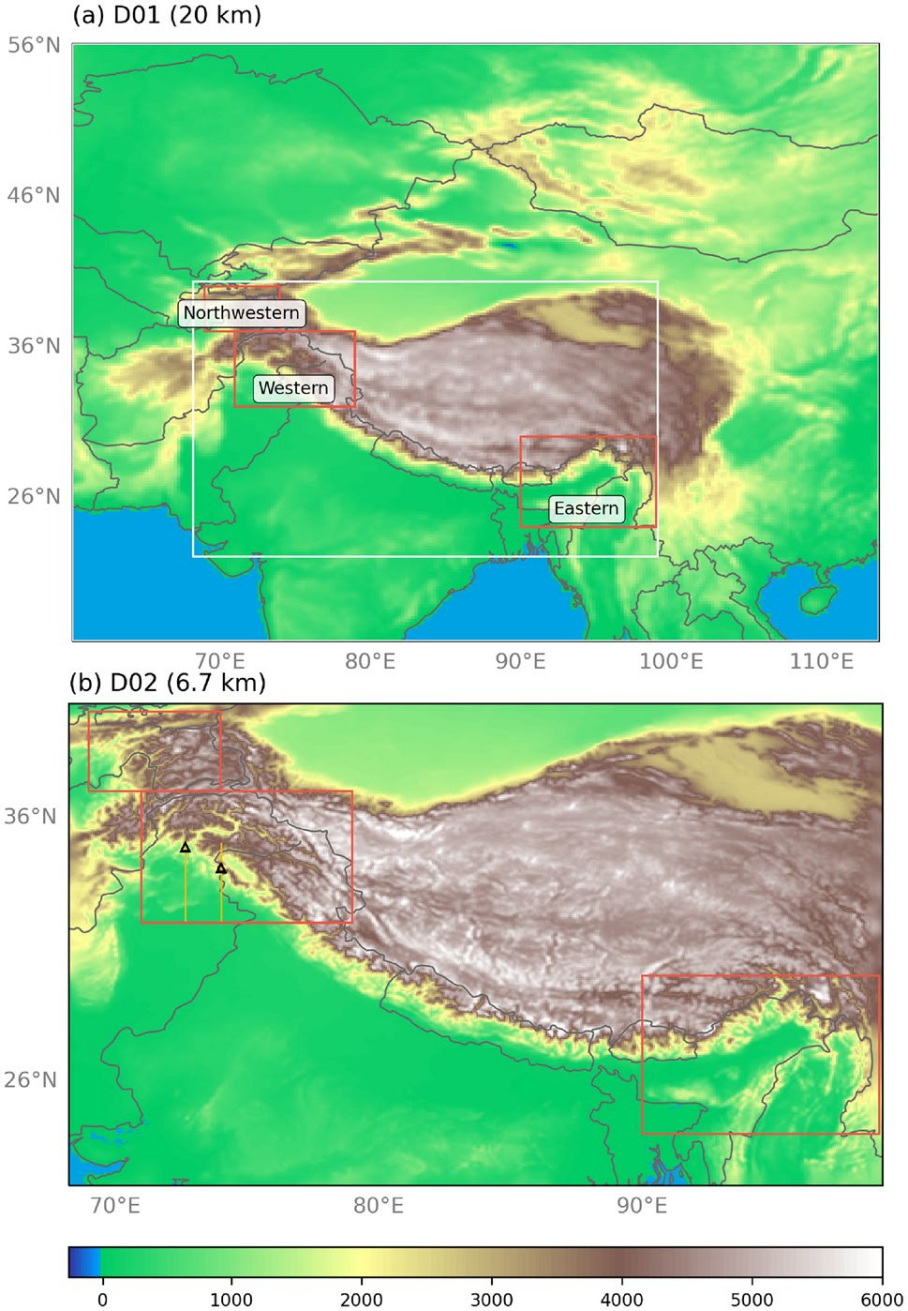
**a** Elevation (shaded; m) in the WRF model outer domain (D01) for downscaling to 20 km grid spacing resolution. The white box indicates the extent of the inner domain. The red boxes indicate the location of above-average precipitation during the Northwestern, Western, and Eastern HMA AR Types and are used as subregions for analysis. **b** Elevation (shaded; m) in the WRF model inner domain (D02) for downscaling to 6.7 km grid spacing resolution. The red boxes are the same as in (**a**). The yellow lines indicate the location of the vertical cross-sections in Figs. 9 and 10. The black triangles indicate the location of the vertical diagrams in Fig. 11

This study uses 36 years of Climate Forecast System Reanalysis (CFSR) (Saha et al. 2010) dynamically downscaled over HMA to 20 km and 6.7 km spatial resolution and 3-hourly temporal resolution using WRF model version 3.7.1 (Skamarock et al. 2008) described in Norris et al. (2019). This data set extends from April 1979 to March 2015, and each year was run continuously from the beginning of March through the end of March the following year (e.g., from 1 March 1979 00:00 UTC to 1 April 1980 00:00 UTC) to capture the full winter and summer seasons. The first month of every year was discarded due to model spin-up, retaining 12 months per year of simulation. The simulations were performed for two domains with one-way interaction (i.e., no feedback and the inner domain did not affect the outer domain) for 20 km and 6.7 km (3:1 ratio), mapped with a Mercator projection (see Fig. 1 for a map of the domains). There were 50 vertical levels in the simulations from the surface to 50 hPa. Spectral nudging of zonal wave numbers 1–5 and meridional wave numbers 1–4 was applied to all vertical levels in the outer domain for temperature, winds, and geopotential height (Stauffer and Seaman 1990; Stauffer et al. 1991). The physics options for the WRF simulations included the Thompson microphysics scheme (Thompson et al. 2008), fifth-generation Pennsylvania State University-National Center for Atmospheric Research Mesoscale Model (MM5) surface layer scheme (Monin and Obukhov 1954), the Noah-MP (multi-physics) land surface model (Niu et al. 2011), the Rapid Radiative Transfer Model for GCMs (RRTMG) scheme for long-wave and short-wave radiation (Iacono et al. 2008), the Yonsei University boundary layer turbulence transfer scheme (Hong et al. 2006), and Kain-Fritch cumulus and shallow convection scheme on the outer domain only (Kain 2004). For more information on the WRF model configuration for the 6.7 km simulations, please see Norris et al. (2017) and Norris et al. (2019). The accuracy of daily winter rain and snow in the 6.7 km simulations were evaluated against satellite cloud-cover data from MODIS and station observations in Norris et al. (2017). They found that station measurements and WRF were well correlated for daily precipitation at elevations below 3 km, which is where the freezing level typically lies, and WRF is accurate in the timing of winter storms on windward slopes. The same study demonstrated that the 6.7 km grid spacing was sufficient for simulating winter precipitation patterns, and additional downscaling to 2.2 km did not dramatically improve simulated spatio-temporal precipitation patterns.

### Observations and gridded estimates

The European Centre for Medium-Range Weather Forecasts (ECMWF) atmospheric reanalysis of the global climate (ERA5) was used at a 6-hourly and $0.25^{\circ }$ resolutions to explore 250 hPa geopotential heights and IVT for the AR events at the synoptic scale because the WRF 20 km outer domain did not cover the spatial extent of the AR cases (Hersbach et al. 2018a, b, 2020).

Kirschbaum et al. (2010) created a landslide catalog where they identified global landslides that were associated with extreme precipitation. Using this, we examined landslides that occurred within 20–45^∘^N and 65–100^∘^E, and considered a landslide associated with an AR if the footprint of the AR occurred in the same space and time as a landslide.

## Methods

HMA AR events were identified on the days when the tARgetv3 detection algorithm (see Sect. 2.1) identified an AR at elevations greater than 1000 m between 20^∘^N and 40^∘^N and 65^∘^E and 97^∘^E between 1979 and 2015. The ARs were then classified as either a Western, Northwestern, or Eastern HMA AR according to the criteria developed in Nash et al. (2021), where combined Empirical Orthogonal Function and k-means clustering was applied to meridional and zonal IVT (see Nash et al. 2021 for more details on identifying HMA ARs). Each HMA AR Type results in above-average precipitation in the area for which they were named and include northwestern HMA (66–74^∘^E and 37–40^∘^N), western HMA (71–79^∘^E and 32–37^∘^N), and eastern Himalaya (90–100^∘^E and 24–30^∘^N) (see Fig. 1 for subregion extents).

This study evaluates how thermodynamic characteristics during HMA ARs have changed in recent decades. To do so, we used the 20 and 6.7 km WRF simulations to examine trends in the freezing level and integrated vapor transport (IVT) during Northwestern, Western, and Eastern HMA ARs during the winter months between 1979 and 2015. The nonparametric Mann-Kendall test was used to identify and plot significant trends for the months of December, January, and February (DJF) over HMA.

To calculate the height of the freezing level, we reverse interpolated WRF temperature (^∘^C) to find the geopotential height (m) of the 0 ^∘^C isotherm (Harris et al. 2000). We used the lowest geopotential height in the case of multiple 0 ^∘^C isotherms (i.e., temperature inversions). In the case where the temperature was less than 0 ^∘^C throughout the entire column (e.g., higher elevation locations), the freezing level was flagged as missing. We classified each HMA AR day as either below- or above-average freezing level based on the average conditions within its respective subregion (see Fig. 1b for subregion extents). To classify AR days according to the height of the freezing level, we followed two steps. First, we identified the area with above-average precipitation during each HMA AR type (e.g., Northwestern, Western, and Eastern). Second, we calculated the average freezing level for that region, separately for each AR type for the duration of the AR event standardized by the AR climatological mean. For example, from 13 to 15 January 1981, there was a Western HMA AR and within the area where above-average precipitation usually occurs during Western HMA ARs (71–79^∘^E and 32–37^∘^N), the average height of the standardized freezing level anomaly was greater than 0. Therefore, we classified that HMA AR as an event with above-average freezing level. Below-average freezing level ARs were classified where the average height of the standardized freezing level anomaly was less than 0. This was repeated for all HMA ARs between 1979 and 2015.

IVT, a variable widely used in the detection and characterization of ARs (Guan and Waliser 2015; Nash et al. 2021; Rutz et al. 2014), was calculated by taking the 3-hourly model data, interpolating u and v wind components (m s^-1^), and water vapor mixing ratio (kg kg^-1^) between 300 hPa and the surface (see Appendix A for equations). We also examined the vertical fluxes of water vapor, or the water vapor flux at each pressure level, to identify the height at which the majority of the moisture is located within each AR. At each pressure level, water vapor flux in the v direction is calculated by multiplying the v component wind and specific humidity (q) (same for u direction, but with u component wind) (see Appendix B for equations).

For precipitation-related variables (e.g., rain, snow, precipitation), model data from every three hours at 00:00 UTC, 03:00 UTC, 06:00 UTC, 09:00 UTC, 12:00 UTC, 15:00 UTC, 18:00 UTC, and 21:00 UTC provided the accumulation from the initialization date of each year. This was then used to compute the mean values of precipitation for time (t) by subtracting precipitation at time (t), from precipitation at time (t+1). We also computed the fraction of frozen precipitation by taking frozen precipitation types (e.g., snow, ice, and graupel) and calculating their overall fraction to total precipitation.

To determine the threshold for extreme AR events, we evaluated two metrics within the subregion of the given HMA AR Type (e.g., Northwestern, Western, or Eastern—see Fig. 1b for subregion extents) for the duration of the AR event: (1) area-maximum precipitation and (2) area-maximum IVT. Area-maximum precipitation for the duration of the AR event gave the clearest picture of how IVT influences precipitation during HMA ARs. We then considered an AR event extreme if IVT and precipitation exceeded the 85th percentile for that type of AR (e.g., Northwestern, Western, or Eastern).

For variables such as meridional and zonal wind, as well as geopotential heights, the model data was interpolated to the given pressure level. Equivalent potential temperature, specific humidity, u, v, and w wind components were used in vertical cross-section analysis, where we interpolated the data to height above ground level along the identified cross-section.

## Results

### Trend analysis

#### AR frequency and intensity trends

**Fig. 2:**
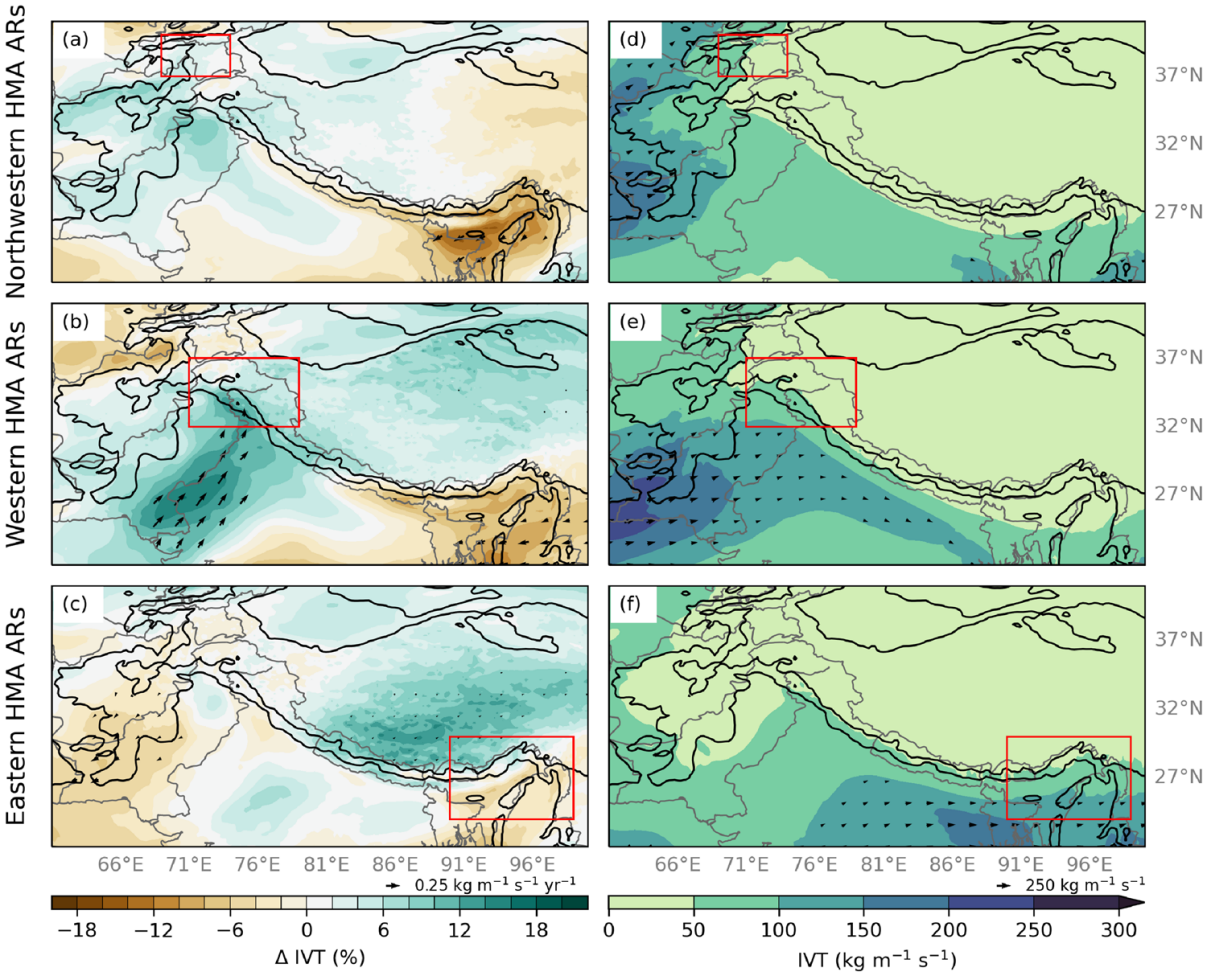
**a** Winter (DJF) seasonal trends in integrated vapor transport (IVT, shaded; %) for Northwestern HMA AR days based on 20 km WRF data for 1979–2015. IVT vectors indicate trends that are considered statistically significant at the 5% significance level. The black contours are the location of 1- and 3-km elevation. The red box indicates the extent of Northwestern HMA. **b** Same as (**a**) but for Western HMA ARs. **c** Same as (**a**) but for Eastern HMA ARs. **d** The average IVT (shaded; kg m^-1^ s^-1^) and IVT direction and magnitude (vectors; kg m^-1^ s^-1^) for DJF Northwestern HMA ARs between 1979–2019 using 20 km WRF data. **e** Same as (**d**) but for Western HMA ARs. **f** Same as (**d**) but for Eastern HMA ARs

To characterize recent changes in HMA ARs, we examined seasonal trends in AR-related IVT using the dynamically downscaled WRF data. We found no significant trends in the frequency of the three HMA AR Types, nor trends in IVT for non-AR days. Figure 2 shows the percent change in IVT since 1979 during days considered to have a HMA AR. During Western HMA ARs, IVT has significantly increased across northwest India and much of Pakistan by 16% (0.3 kg m^-1^ s^-1^ yr^-1^) (see Fig. 2b). Figure 2a, b show that IVT has decreased 12–16% in eastern HMA ($-$0.2 kg m^-1^ s^-1^ yr^-1^) during Northwestern and Western HMA ARs.

#### Freezing level trends

**Fig. 3:**
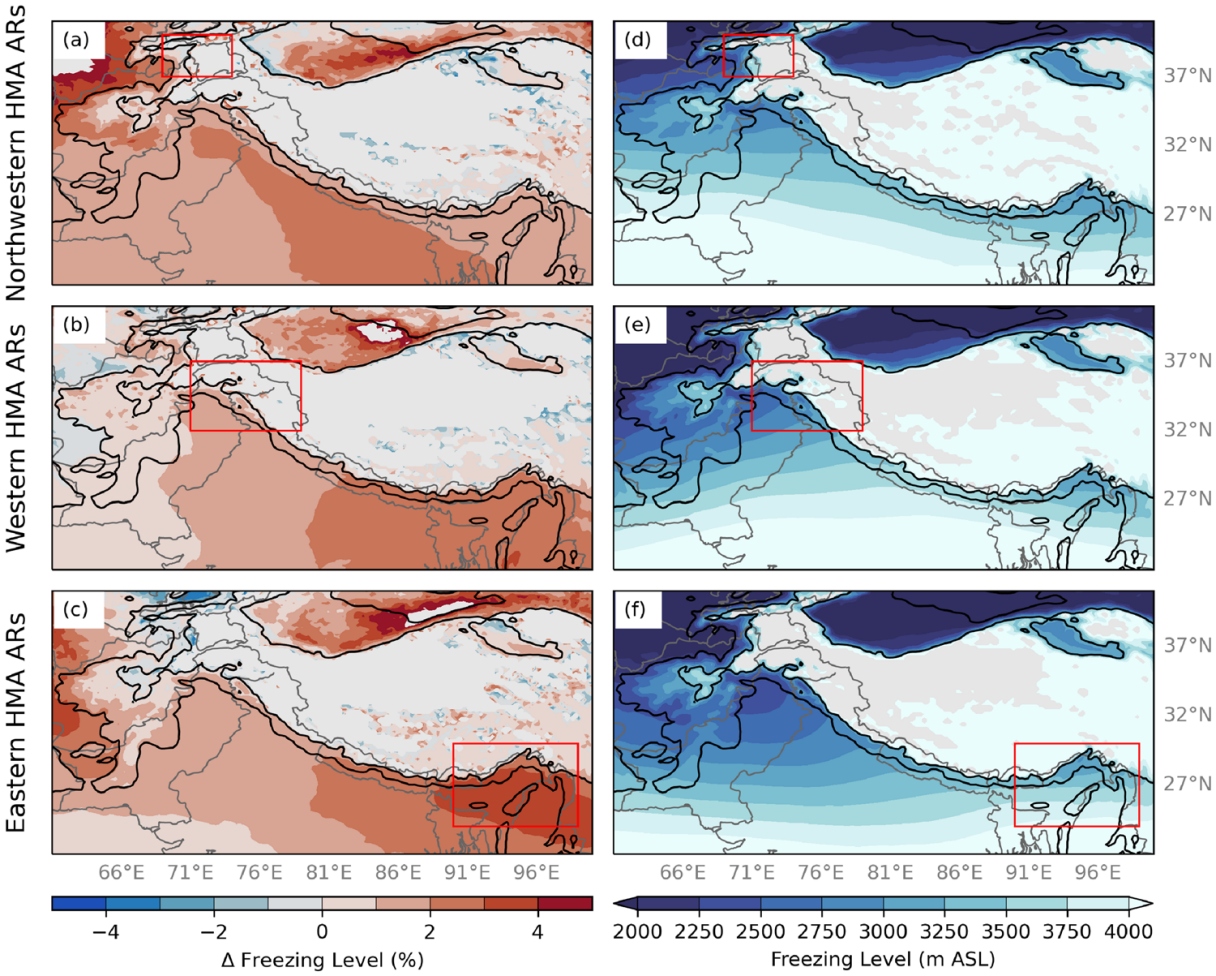
**a** Winter (DJF) seasonal trends in the freezing level (shaded; %) for Northwestern HMA AR days based on 20 km WRF data for 1979–2015. Data are only plotted where trends are considered statistically significant at the 10% significance level. The black contours are the location of 1- and 3-km elevation. The red box indicates the extent of Northwestern HMA. **b** Same as (**a**) but for Western HMA ARs. **c** Same as (**a**) but for Eastern HMA ARs. **d** The average freezing level (shaded; m ASL) for DJF Northwestern HMA ARs between 1979–2019 using 20 km WRF data. **e** Same as (**d**) but for Western HMA ARs. **f** Same as (**d**) but for Eastern HMA ARs

The freezing level influences the altitude to which rain falls, and the resulting fraction of frozen precipitation (Guan et al. 2016). The freezing level varies as a function of elevation and latitude across HMA. For example, between 1 and 3 km in HMA, the average height of the freezing level is between 2.5 and 3.5 km, while elevations higher than 3 km have an average freezing level of 4 km or greater. Between 25^∘^N and 30^∘^N, the average height of the freezing level is between 3 and 3.5 km, whereas poleward of 30^∘^N, the average height is below 3 km. However, an upper-level cut-off low associated with high amplitude troughs can decrease the freezing level for the duration of a storm, increasing the fraction of frozen precipitation relative to the total precipitation. On the other hand, ARs often increase the freezing level, resulting in more rain and less snow falling at higher altitudes (Guan et al. 2016; Neiman et al. 2008, 2011). During HMA ARs, the freezing level across India has significantly increased ($p < 0.1$) between 1979 and 2015 (Fig. 3). Nonetheless, the magnitude of the increase of the freezing level varies by AR type. For example, during Western HMA ARs (Fig. 3b), the freezing level increased by 2% (1 m yr^-1^) across HMA foothills, while during Northwestern and Eastern HMA ARs (Fig. 3a, c), the freezing level increased by 3–4% (4 m yr^-1^) in northwestern and eastern HMA foothills. Due to large amounts of missing data in higher elevations, results ± 5 m yr^-1^ were omitted. Overall, the freezing level has significantly increased across HMA during all three AR types between 1979 and 2015. When considering non-AR days, trends in the freezing level in northwestern and western HMA were not significant, indicating the importance of the relationship between the freezing level and ARs.

**Fig. 4:**
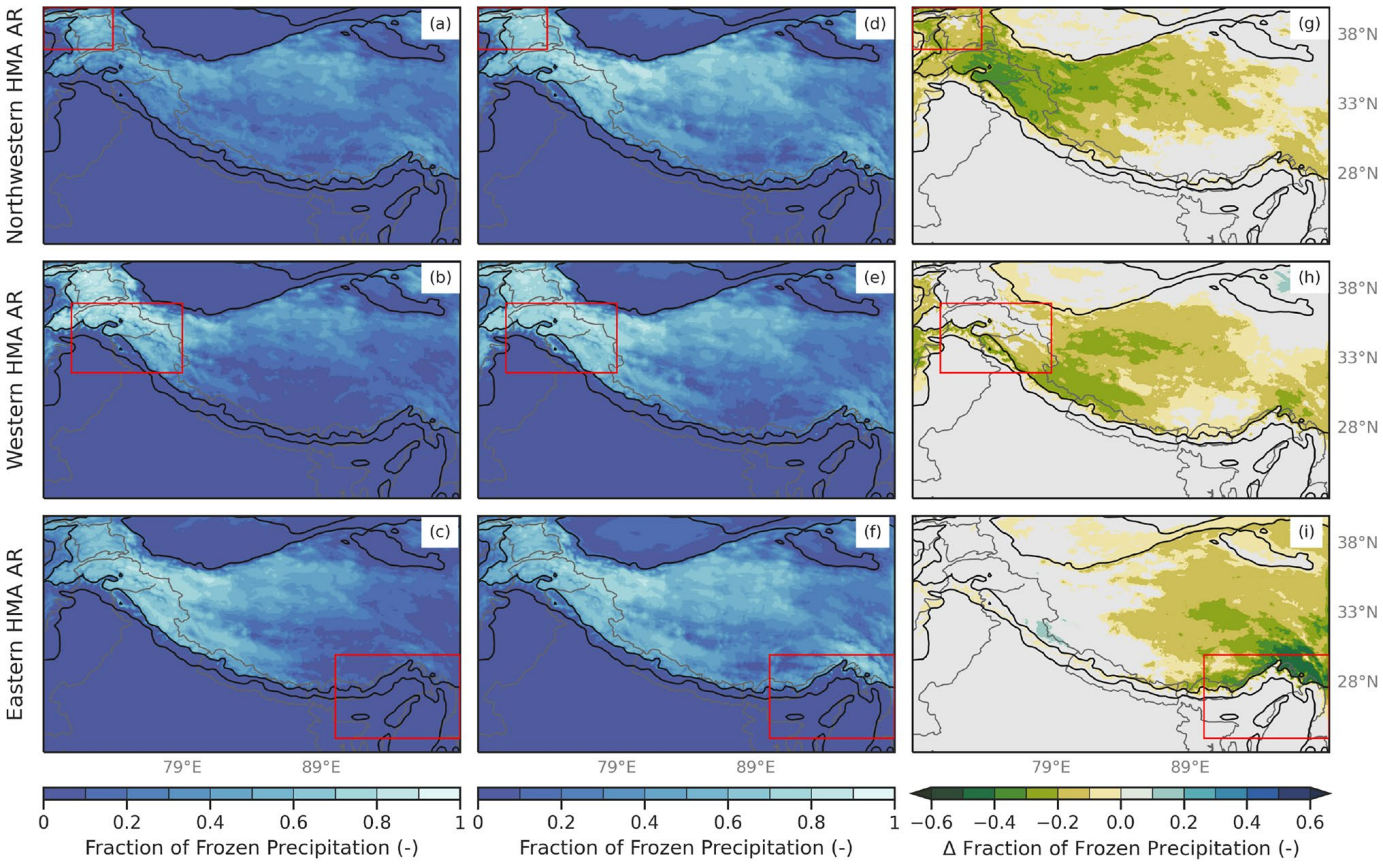
**a** WRF 6.7 km fraction of frozen precipitation (shaded; -) for Northwestern HMA ARs above-average freezing level conditions within the red box. **b** Same as (**a**) but for Western HMA ARs. **c** Same as (**a**) but for Eastern HMA ARs. **d** WRF 6.7 km fraction of frozen precipitation (shaded; -) for Northwestern HMA ARs below-average freezing level conditions within the red box. **e** Same as (**d**) but for Western HMA ARs. **f** Same as (**d**) but for Eastern HMA ARs. **h** Composite differences of WRF 6.7 km fraction of frozen precipitation (shaded; -) for Northwestern HMA ARs above-average freezing level conditions and below-average freezing level conditions within the red box. Only differences (above-average conditions minus below-average conditions) in the fraction of frozen precipitation that are considered at or above the 95% confidence level are shaded. **i** Same as (**h**) but for Western HMA ARs. **j** Same as (**h**) but for Eastern HMA ARs

To investigate the influence of the increased moisture transport and higher freezing levels on precipitation during HMA AR days, we compared the fraction of frozen precipitation in HMA during above-average freezing level conditions and below-average freezing level conditions. Composites of the difference in the fraction of frozen precipitation that occurs between above- and below-average freezing level heights, for each AR type, are shown in Fig. 4. During HMA AR days with above-average freezing level conditions, the fraction of frozen precipitation was significantly less than HMA AR days with below-average freezing level conditions (Fig. 4). Interestingly, the decreased fraction of frozen precipitation extends beyond the region of above-average precipitation that we typically see during each type of HMA AR. Specifically, the decreased fraction of frozen precipitation extends, and is sometimes maximized, over areas eastward of these. This distribution is expected given that relatively warm air is typically transported poleward along and ahead of ARs, while along the AR, heavy precipitation may lower the freezing level locally. For example, during Western HMA AR days, there is 10–40% less frozen precipitation during above-average freezing level conditions across western and central HMA compared to below-average freezing level conditions (Fig. 4h). These results indicate the broad influence of ARs on the freezing level and resulting precipitation type across HMA.

### HMA AR characteristics

**Fig. 5:**
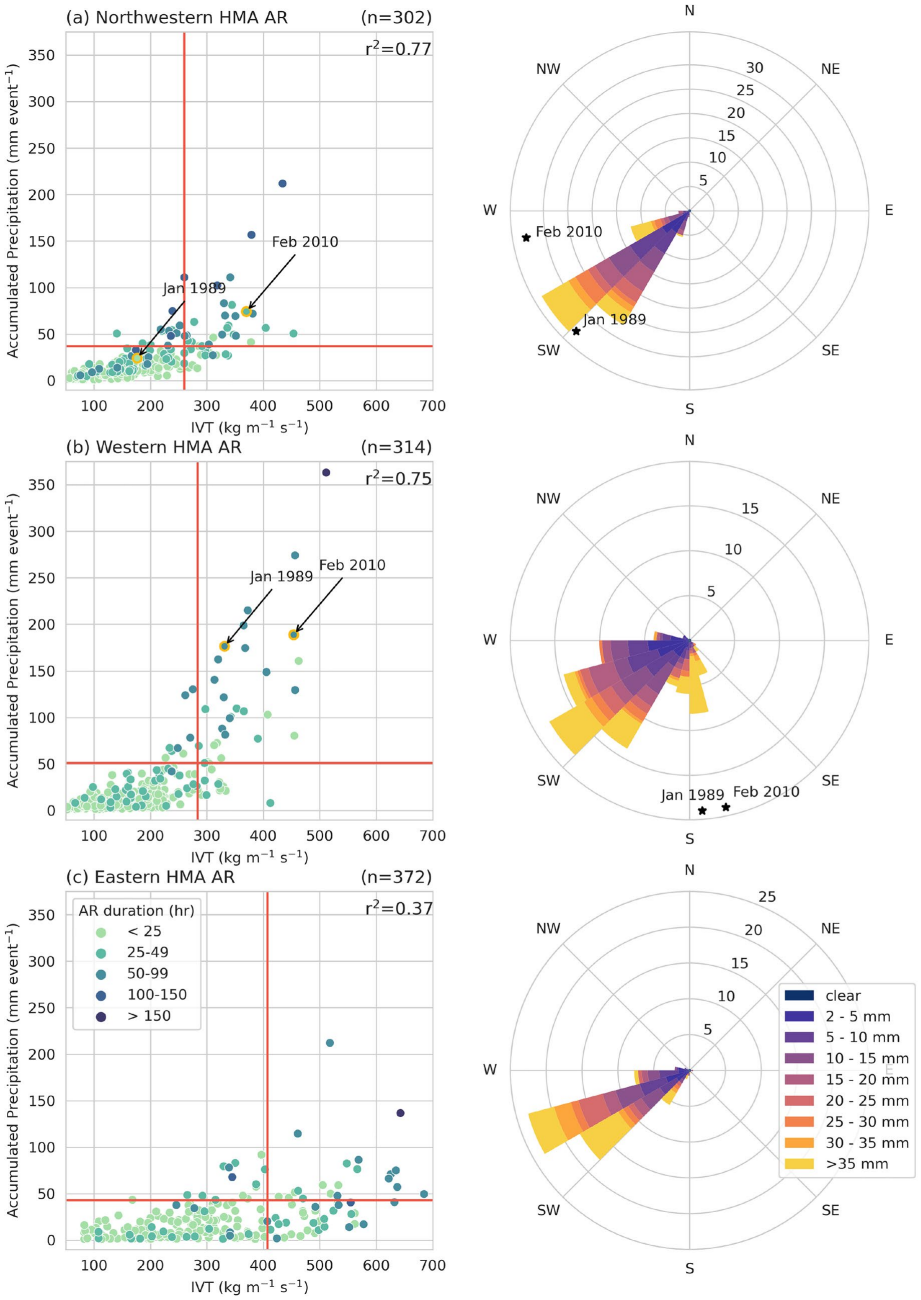
**a** Scatter plot showing ERA-Interim maximum IVT (kg m^-1^ s^-1^) compared to the ERA5 maximum-total precipitation (mm event^-1^) in the region that accumulates above-average precipitation for Northwestern HMA ARs (see Fig. 1b for subregion locations) for the duration of the AR event. The duration (hours) of the AR event is indicated by the shading. The circles with yellow highlight indicate the case studies chosen in Sect. 4.4. The red lines indicate the 85th percentile for IVT (x-axis) and precipitation (y-axis). **b** Same as (**a**) but for Western HMA ARs. **c** Same as (**a**) but for Eastern HMA ARs. **d** Wind rose diagrams for IVT direction from WRF 20 km data for each of the Northwestern HMA AR events ($n = 302$). Total length of each bar indicates the frequency (%) of events with IVT in that particular direction. Length of colored areas within bar indicates the frequency (%) of precipitation in that particular direction. **e** Same as (**d**) but for Western HMA ARs ($n=314$). **f** Same as (**d**) but for Eastern HMA ARs ($n=372$)

Figure 5a–c shows the distribution of maximum IVT and precipitation accumulation for the duration of all HMA AR events within the domain of the given AR type (see Sect. 3 for more details on the methodology). Using multivariate linear regression, we found that IVT magnitude and AR duration explain more than 75% of the precipitation variability during Western and Northwestern HMA ARs and 37% of the precipitation variability during Eastern HMA ARs ($\rho < 0.01$). The former is consistent with observations about ARs that make landfall on the west coast of the US, where a higher IVT magnitude and longer AR duration are typically associated with heavy precipitation AR events that result in floods and landslides (Ralph et al. 2019; Oakley et al. 2017; Rutz et al. 2014). During HMA winter, IVT rarely exceeds 300 kg m^-1^ s^-1^ but can still result in significant precipitation. For Western HMA AR events, only 15% are above the 85th percentile of IVT, but of these, 75% are above the 85th percentile of precipitation, highlighting the importance of IVT in driving heavy precipitation events. Results are similar for Northwestern HMA, but less robust for Eastern HMA. This may be because IVT has significantly decreased 12–16% across eastern HMA during all HMA ARs (Fig. 2). Another AR characteristic that is important to precipitation outcomes is the direction of the IVT relative to the topography. Figure 5d–f shows that most HMA AR IVT was southwesterly. However, almost 10% of Western HMA ARs had southerly IVT and these were almost exclusively very heavy precipitation events (i.e., more than 35 mm of precipitation). These results suggest that regional characteristics such as IVT magnitude, direction, and duration are important when considering short-term predictability (less than 7 days) of these events.

Of the 29 Western HMA AR events with precipitation and IVT above the 85th percentile, 15 occurred during above-average freezing level conditions, 14 occurred during below-average freezing level conditions. Therefore, the remainder of this paper focuses on the synoptic and mesoscale conditions for two Western HMA ARs to determine the underlying mechanisms that contributed to these differing outcomes.

### Case study selection

To better understand the relationship between freezing level, precipitation type, and the potentially increased risk of landslide, we examine the synoptic and mesoscale atmospheric conditions during two distinct AR events: one with an above-average freezing level and one with a below-average freezing level. These two AR events both began as Northwestern HMA ARs and transitioned to Western HMA ARs, both had above-average IVT that resulted in extreme precipitation (above the 85th percentile for precipitation and IVT), but critically, featured very different freezing level distributions.

**Fig. 6:**
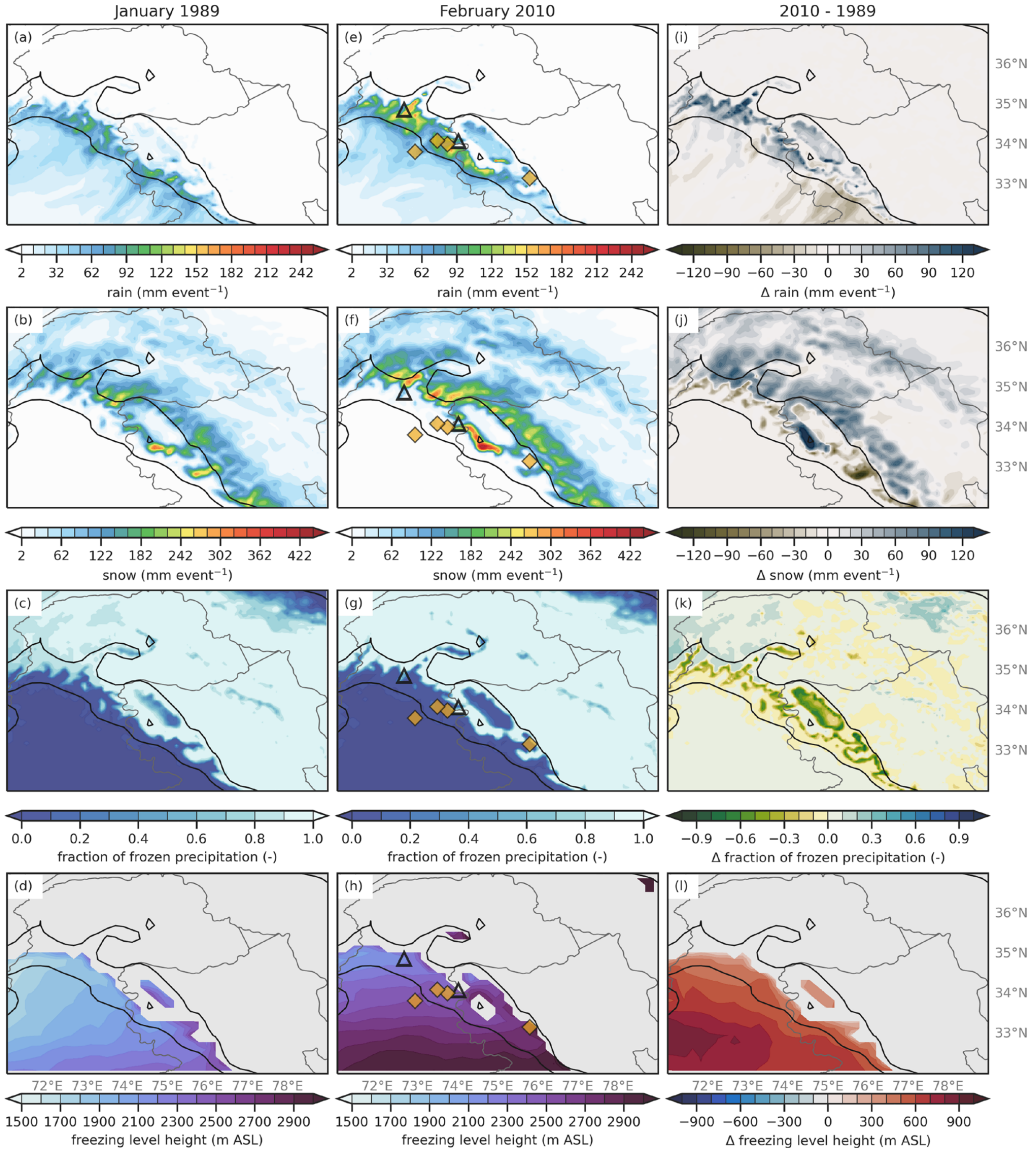
**a** Total event WRF 6.7 km rain (shaded; mm event^-1^) for the January 1989 AR. The black contours are the location of 1- and 3-km elevation. **b** Total event WRF 6.7 km snow (shaded; mm event^-1^) for the January 1989 AR. **c** Average event WRF 6.7 km fraction of frozen precipitation (shaded; -) for the January 1989 AR. **d** Average WRF 20 km freezing level (shaded; m ASL) for the January 1989 AR. **e–h** Same as (**a–d**) but for the February 2010 AR. The yellow diamonds and black triangles indicate the location of a precipitation-triggered landslides during the 2010 AR event, the triangles are the same points in Figs. 1b and 9e,f. **i** The difference in rain (shaded; mm event^-1^) for the February 2010 AR minus the January 1989 AR. **j** Same as (**i**) but for snow (shaded; mm event^-1^). **k** Same as (**i**) but for the fraction of frozen precipitation (shaded; -). **l** Same as (**i**) but for the freezing level (shaded; m ASL)

The below-average freezing level AR case occurred from 3 January 06:00 UTC to 6 January 1989 12:00 UTC (duration of 78 h) and resulted in up to 218 mm of rain and 350 mm of snow in locations across Western HMA, but there were no reported landslides or damages (Fig. 6a, b). The above-average freezing level AR case occurred from 4 February 2010 00:00 UTC to 8 February 2010 18:00 UTC (duration of 90 h) and resulted in around 250 mm of rain and 420 mm of snow in locations across western HMA (Fig. 6e, f). In this case, six precipitation-related landslides occurred between 6 and 8 February and appear to be associated with this AR (locations identified as yellow squares and black triangles in Fig. 6e–h). There were many devastating impacts from these landslides, including at least sixteen deaths, destruction of many houses and buildings, and isolation of many communities due to highway closures and damage (Kirschbaum et al. 2010). Figure 6i–l shows the differences in the total event rain, snow, fraction of frozen precipitation and freezing level between the two AR events. Overall, there was more precipitation during the February 2010 AR, likely due to the longer duration of the 2010 AR compared to the 1989 AR. Section 4.5 discusses other mechanisms for the differences in precipitation.

Focusing on the area where changes in the freezing level are likely to affect the fraction of frozen precipitation (i.e., between 1 and 3 km), the February 2010 AR had more rain, and less snow compared to the January 1989 AR (Fig. 6i, j). At these elevations, the freezing level during the February 2010 event ranged from 0 to 400 m higher than during the January 1989 events, resulting in 10–70% less frozen precipitation over these areas (Fig. 6k, l). Some elevations above 3 km received more snow during the February 2010 event due to the intensity and duration of the AR, combined with temperatures still cold enough for snow at these altitudes.

### Synoptic characteristics of the HMA AR case studies

**Fig. 7:**
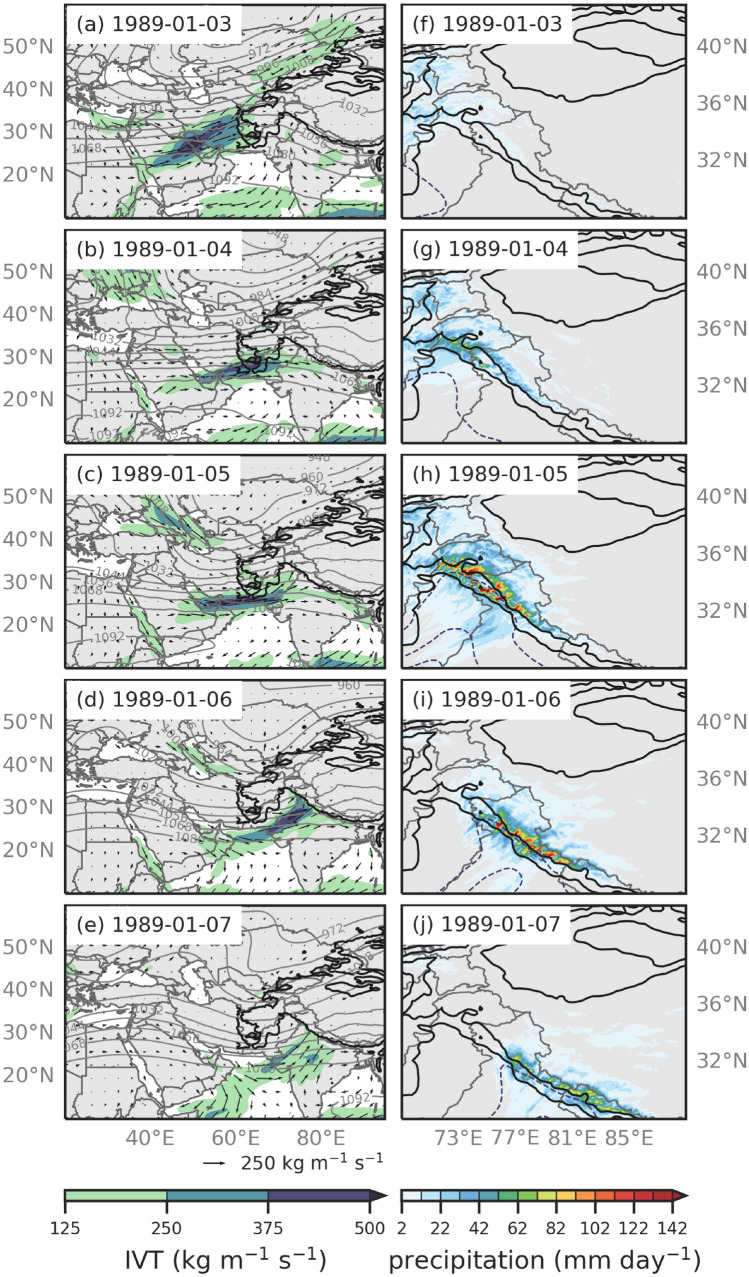
**a** ERA5 IVT (shaded and vectors; kg m^-1^ s^-1^) and 250 hPa geopotential heights (contours; dam) at 3 January 1989 00:00 UTC. The black contour is the location of 1-km elevation. **b–e** Same as (**a**) but for every 24 h between 4 and 7 January 00:00 UTC 1989. **f** WRF 6.7 km precipitation (shaded; mm day^-1^) and IVT (dashed contour; kg m^-1^ s^-1^) at 3 January 1989 00:00 UTC. The black contours are the location of 1- and 3-km elevation. **g–j** Same as (**f**) but for every 24 h 4 to 7 January 00:00 UTC 1989

**Fig. 8:**
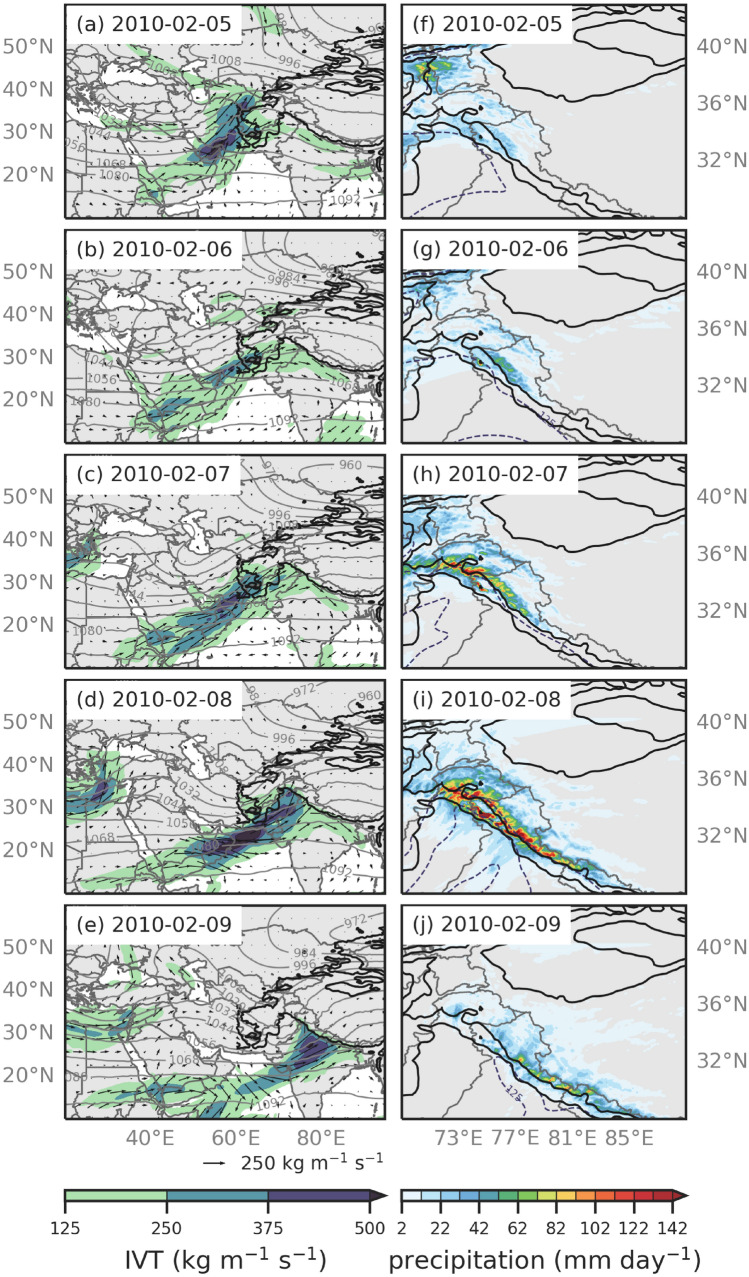
Same as Fig. 7 but for every 24 h between 5 and 9 February 2010 00:00 UTC

Figures 7 and 8 show the ERA5 IVT (kg m^-1^ s^-1^), 250 hPa geopotential heights (dekameters), and the 6.7 km WRF precipitation (mm hour^-1^) every 24 h for the duration of the AR event. ERA5 is used to describe the underlying synoptic conditions of the AR events (e.g., 250 hPa geopotential heights and IVT), as the WRF 20 km outer domain does not cover the large spatial extent of the ARs. When compared to the WRF data, ERA5 IVT and 250 hPa geopotential heights were consistent. Because both AR events were quasi-stationary, 24-hourly analyses were sufficient to capture the large-scale dynamics.

#### January 1989 event

On 3 January 1989 00:00 UTC, a positively tilted upper-level trough centered at 60^∘^E and 50^∘^N transported moisture across Southwest Asia (Fig. 7a). Additionally, an anticyclone formed over the very northern basin of the Arabian Sea, adding to the moisture of the AR all the way through 4 January 00:00 UTC (Fig. 7b). The IVT of this AR was between 375 and 500 kg m^-1^ s^-1^, but was confined to a small core within the AR. On 5 January 00:00 UTC (Fig. 7c), the upper-level trough at 45^∘^N and 60^∘^E helped maintain the southwesterly IVT as it reaches western HMA. Most of the precipitation for this event occurred in western HMA between 5 and 6 January, when the AR direction east of the Hindu Kush transitioned from westerly to southwesterly, orographically forcing moisture directly perpendicular to the topography of western HMA (Fig. 7c, d, h, i). By 7 January 00:00 UTC, the moisture moved to central HMA (Fig. 7e).

#### February 2010 event

On 5 February 2010 00:00 UTC, an upper-level trough centered at about 35^∘^E and 40^∘^N and anticyclonic flow centered over the Arabian Sea transported moisture within the AR across Southwest Asia (Fig. 8a). The southwesterly moisture in the core of the AR peaked around 500 kg m^-1^ s^-1^ and reached the Hindu Kush, Pamirs, and Tien Shan ranges. Precipitation occurred where the moisture from the AR reached these high elevation regions (Fig. 8a, f). By 6 February (Fig. 8b), the upper-level trough shifted to 40^∘^E and 40^∘^N, the anticyclone over the Arabian Sea remained in place, and the moisture from the AR was funneled into northwest HMA, western HMA, and central Himalayas, decreasing IVT within the AR to less than 375 kg m^-1^ s^-1^. At this time, precipitation was roughly 3 mm hr^-1^. On 7 February 2010 00:00 UTC (Fig. 8c), the anticyclone over the Arabian Sea shifted eastward and remained directly over India for the next few days. Anticyclonic circulation around this feature potentially added moisture from the Bay of Bengal and Arabian Seas to the AR, as peak IVT in the core of the AR reached 500 kg m^-1^ s^-1^ and the precipitation rate increased across the Hindu Kush and western HMA to above 7 mm per hour (Fig. 8h). On 8 February, 00:00 UTC, a new anticyclonic circulation that appeared centered at 50^∘^E and 10^∘^N (Fig. 8d) provided additional moisture for the AR from the Arabian Sea, but the relatively stationary midlatitude conditions (i.e., upper-level trough centered at 60^∘^E and upper-level ridge centered at 80^∘^E) aided in pulling the moisture all the way to HMA for such an extended period. The precipitation in western HMA was at its most intense during 8 February, when the orientation of the AR was perpendicular to the topography, reaching precipitation rates greater than 10 mm hr^-1^ (Fig. 8i). By 9 February 00:00 UTC, the upper-level trough and ridge shifted eastward and weakened so that the AR began to quickly dissipate (Fig. 8e).

Both events show that most of the precipitation falls in western HMA when the IVT becomes terrain locked in the “notch” or area that is enclosed on three sides by high elevation mountains. Lang and Barros (2004) originally identified this “notch” when they demonstrated that the terrain in western HMA, combined with the location of the subtropical jet, increases the likelihood that an extratropical cyclone remains quasi-stationary in this area, increasing the orographic precipitation in western HMA. The synoptic and precipitation patterns during both our AR events are consistent with the 10 January 1999 extreme precipitation event, described in Norris et al. (2015) as a western notch pattern, and classified in Nash et al. (2021) as a Western HMA AR type. This demonstrates the importance of properly classifying HMA AR types to identify the impacts of ARs on orographic precipitation and potential hazards.

### Mesoscale characteristics of the HMA AR case studies

**Fig. 9:**
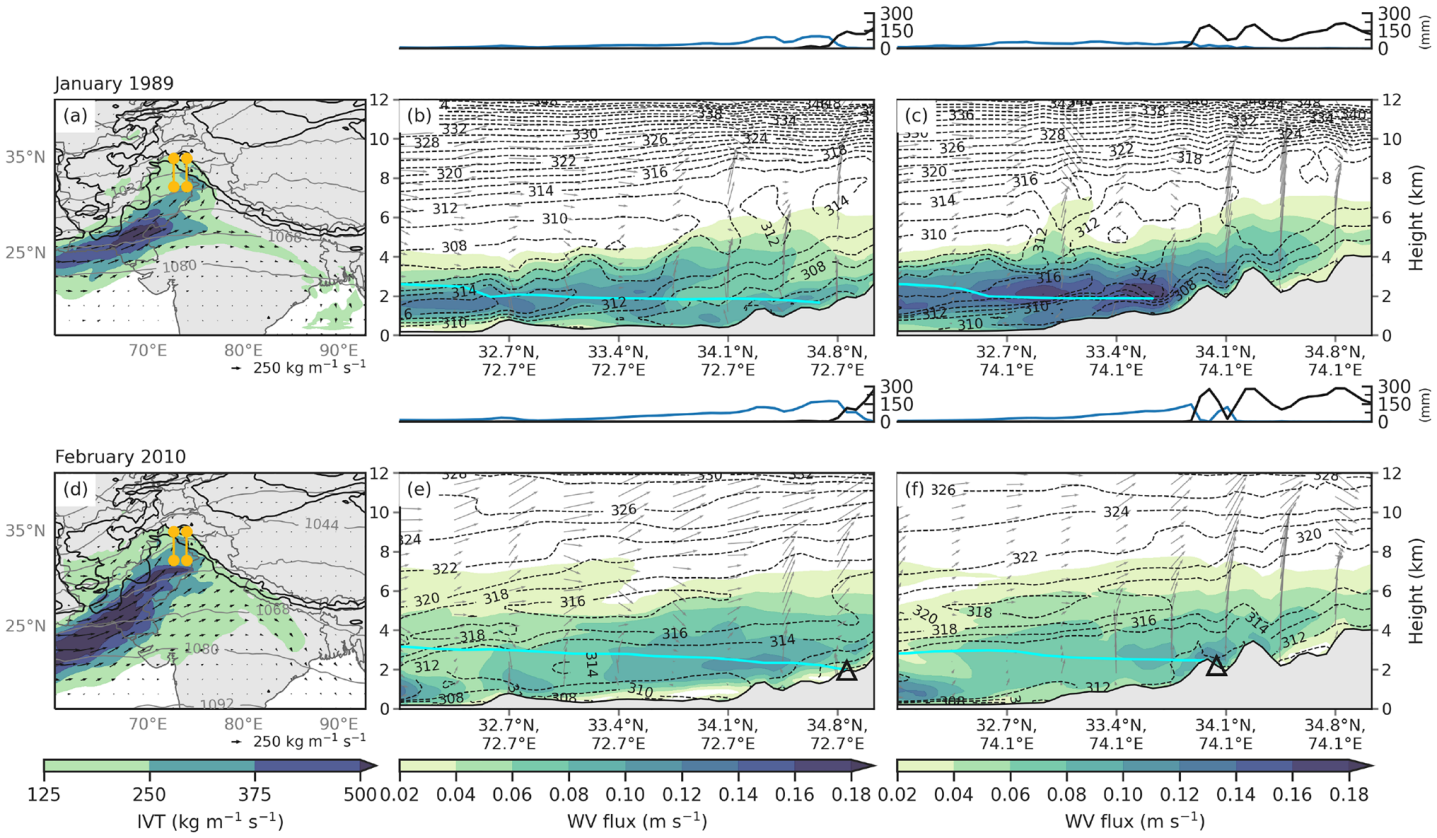
**a** WRF 20 km IVT (shaded and vectors; kg m^-1^ s^-1^) on 5 January 1989 12:00 UTC. The black contours are the location of 1- and 3-km elevation. The left yellow line indicates the cross-section for (**b**), and the right yellow line is the cross-section (**c**). **b, c** WRF 6.7 km water vapor flux (shaded; m s^-1^), $\theta _{E}$ (dashed contour; K), and vertical velocity (vectors; m s^-1^) interpolated to height above ground level (km). The cyan line is the freezing level. The line plot above is WRF 6.7 km event-total rain (mm; blue line) and snow (mm; black line) along the same cross-section. **d–f** Same as (**a–c**) but for 8 February 2010 00:00 UTC. The black triangles indicate the points of two of the six landslides triggered during this event and are also shown in Figs. 1b and 6e–h

**Fig. 10:**
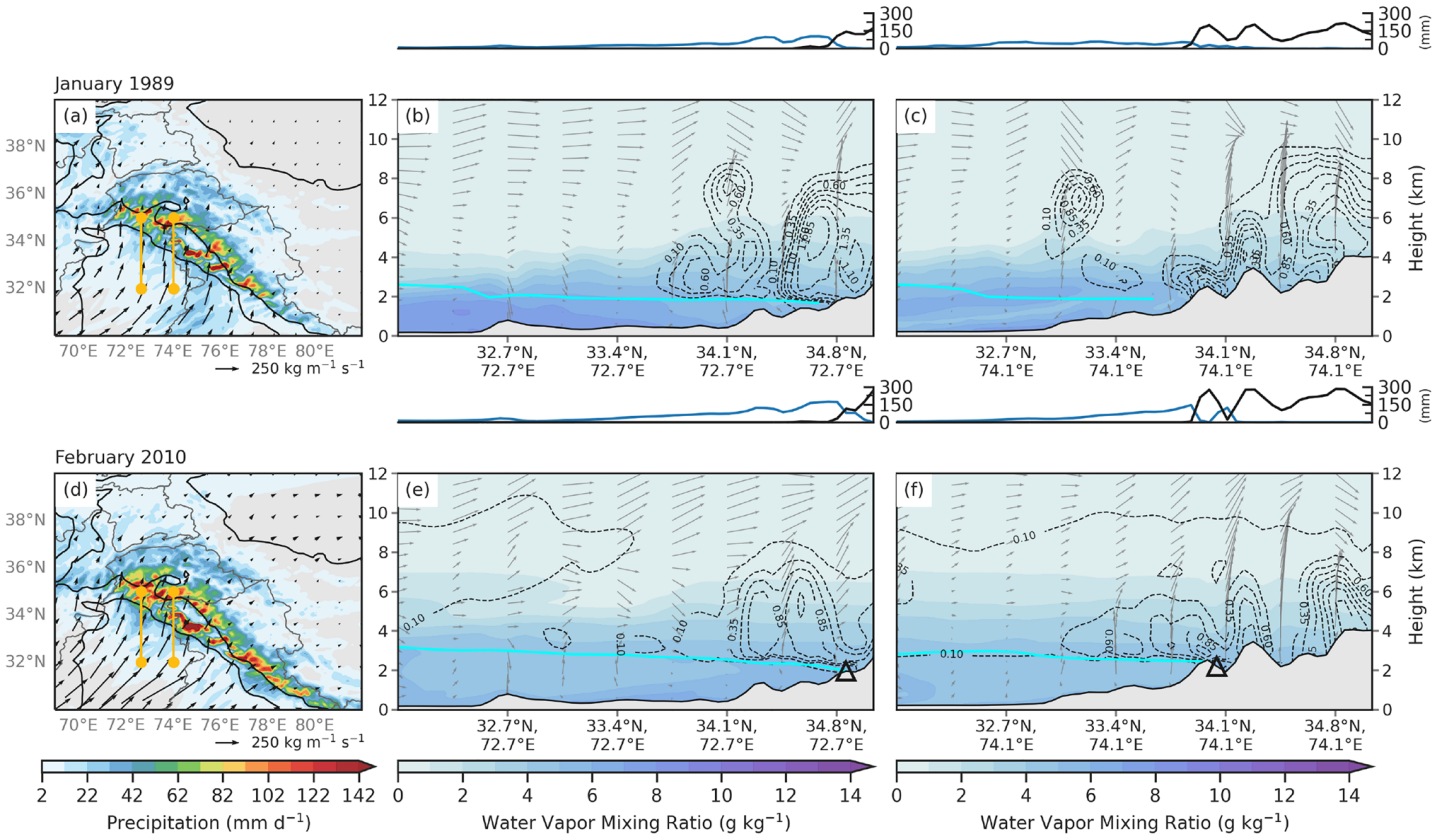
**a** WRF 6.7 km precipitation (shaded; mm day^-1^) and IVT (vector; kg m^-1^ s^-1^) on 5 January 1989 12:00 UTC. The black contours are the location of 1- and 3-km elevation. The left yellow line indicates the cross-section for (**b**), and the right yellow line is the cross-section (**c**). **b, c** WRF 6.7 km snow water mixing ratio (contour; g kg^-1^), water vapor mixing ratios (shaded; g kg^-1^), and vertical velocity (vectors; m s^-1^) interpolated to height above ground level (km). The cyan line is the freezing level. The line plot above is WRF 6.7 km event-total rain (mm; blue line) and snow (mm; black line) along the same cross-section. **d–f** Same as (**a–c**) but for 8 February 2010 00:00 UTC. The black triangles indicate the points of two of the six landslides triggered during this event and are also shown in Figs. 1b and 6e–h

To better understand the underlying mechanisms driving different orographic precipitation amounts and types (i.e., snow, rain) in western HMA, we examined the mesoscale conditions for both AR events. Precipitation in AR events primarily results from a combination of orographic lift, moisture transport, and instability. To further examine these processes in the WRF simulations, we show along AR cross-sections (see yellow lines in Figs. 9a, d and 10a, d), showing the horizontal water vapor flux (m s^-1^), equivalent potential temperature contours (K), snow-water mixing ratio (g kg^-1^), water vapor mixing ratio (g kg^-1^), and vertical velocity (m s^-1^) interpolated to height above ground level (km) at 5 January 1989 12:00 UTC and 8 February 2010 00:00 UTC. To facilitate a simple comparison, we focused on the day of peak IVT for each event, since the results for other times during each event exhibited similar features.

On 5 January 1989 12:00 UTC, the flow of IVT was zonal as it crossed the northern basin of the Arabian Sea, and then turned into more of a southwesterly flow in western HMA, getting caught in the western notch (Figs. 9a and 10a). The western cross-section (Fig. 9b) shows that the moisture flux within the AR south of 32.7^∘^N extended from the surface to 4 km, peaking at 0.12 m s^-1^ below 2 km. The moisture in the AR followed the isentropes as they gently sloped upward between 32.7^∘^N and 34.1^∘^N. The below-average freezing level (2 km) kept the snow-water mixing ratio below 0.6 g kg^-1^ south of 34.1^∘^N (Fig. 10b). All the precipitation south of 34.8^∘^N fell as rain and transitioned to snow where the freezing level intersected with the topography (Fig. 9b). The eastern cross-section (Fig. 9c) shows moisture flux exceeding 0.18 m s^-1^ between 3 and 4 km south of 34.1^∘^N. There was a peak in snowfall that coincided with the first mountain peak taller than 2 km and snow water mixing ratio up to 1.9 g kg^-1^ (Fig. 10c), north of 33.4^∘^N implying that the moisture was forced orographically. Precipitation in the eastern cross-section just north of 34.1^∘^N fell as snow, while falling as rain south of that location. Like the western cross-section, the transition from rain to snow occurred near and upstream of where the freezing level intersected with the topography, consistent with evaporative cooling and other snow-level lowering processes along windward slopes (Minder et al. 2011).

**Fig. 11:**
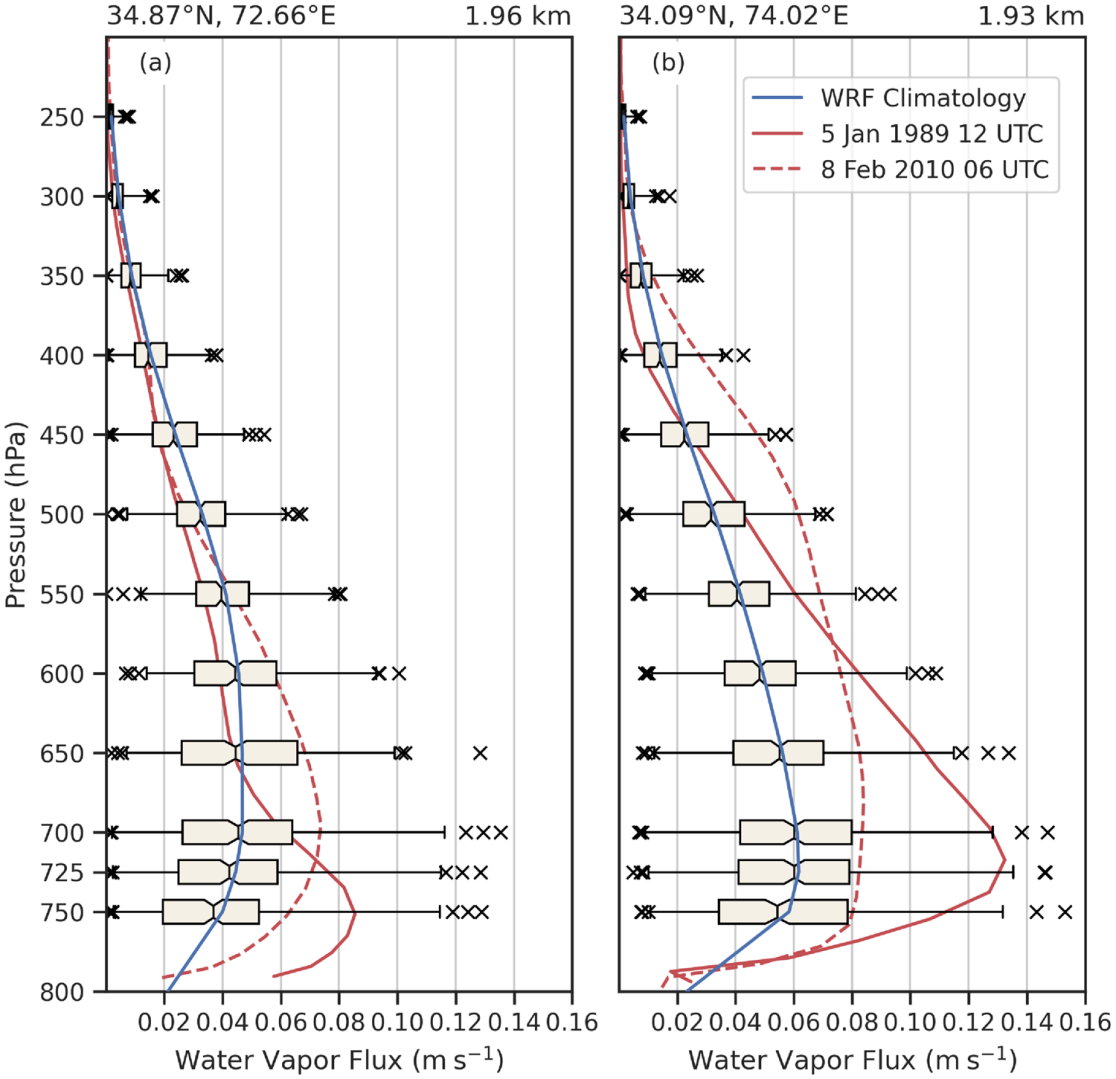
**a** Climatological vertical profile of horizontal water vapor flux (m s^-1^) based on WRF at 34.87^∘^N, 72.66^∘^E for all days when AR conditions are met during the months of December, January, or February between 1979 and 2015 at this location (blue line and box-and-whisker plots show the distribution of the 284 events), and vertical profile of horizontal water vapor flux (m s^-1^) based on WRF at the same location on 5 January 1989 12:00 UTC (red solid line) and 8 February 2010 06:00 UTC (red dashed line). The box extends from lower to upper quartiles of the data, with a black line at the mean. The whiskers show the range of the data from the 5th percentile to the 95th percentile, and outliers are shown as points past the end of the whiskers. **b** Same as (**a**) but for 34.09^∘^N and 74.02^∘^E. The locations of both points are identified by the black triangles in Figs. 1b, 6e–h, 9e,f, and 10e,f

The westernmost cross-section for the February 2010 case (Fig. 9e) shows that the February AR had a deep moist layer, that extended all the way up to 7 km in height, with a maximum of water vapor flux at 0.12 m s^-1^ around 3 km in elevation just north of 34.1^∘^N. Compared to the climatological vertical profile of water vapor flux from the past AR events that reached western HMA (peak of 0.4 m s^-1^ at 500 hPa), moisture within the February 2010 AR was above-average (Fig. 11). Moisture located above the freezing level and the intersection of the freezing level with topography aligned with the peak in snow just south of 34.8^∘^N where snow-water mixing ratio reached 0.85 g kg^-1^ (Figs. 9e and 10e). It is around the location of the transition from rainfall to snowfall where one of the landslides occurred on 8 February 2010 at 72.7^∘^E, 34.8^∘^N, unsurprising with 130 mm of rain and 85 mm of snow, most of which fell in under 24 h.

Further east, the deep, moist layer of the AR persisted, although there was a smaller core of moisture flux that reached 0.12 m s^-1^ (Fig. 9f). Snow-water mixing ratio within this moisture aloft was around 0.6 g kg^-1^ and water vapor mixing ratio was below 4 g kg^-1^ (Fig. 10f). Orographic lift of the moisture resulted in two peaks of snow, one south and one north of 34.1^∘^N (Fig. 9f). The landslide occurred on the lee side of the slope in the eastern cross-section where moisture flux was highest, and a 60 mm of rain fell in addition to 90 mm of snow (Figs. 9f and 10f).

Similarities between the two events include the synoptic forcing, IVT direction, and average event-total precipitation. In both cases, IVT exceeding 250 kg m^-1^ s^-1^ in the western Himalayas indicated that the moisture became terrain locked in the western HMA notch, resulting in greater than 150 mm precipitation totals in some locations (Fig. 10a, d). Differences between the two events include slightly higher precipitation totals during the February 2010 AR, likely due to the difference in the duration between the two events. The February 2010 AR remained stationary between 6 and 8 February 2010 while the 1989 AR traversed the area from 4 to 6 January 1989. Another reason precipitation rates may have been higher during the February 2010 AR is because the orientation of the peak IVT at the 1 km elevation line was southwesterly, and directly perpendicular to the terrain, while the moisture during the January 1989 AR was southerly (Fig. 10a, d).

Another similarity was that both cases saw rainfall in the foothills, which transitioned to snow once the elevation was above the freezing level. However, between 1 and 3 km elevation, there was almost 150 mm more rain and 200 mm less snow during the February 2010 AR compared to the January 1989 AR (Fig. 6i, j). At 34.8^∘^N and 72.7^∘^E, snow mixing ratio was thirteen times as high during the January 1989 AR (1.35 g kg^-1^) compared to the February 2010 AR (0.10 g kg^-1^) (Fig. 10b, e). The freezing level in the February 2010 AR was 50–600 m higher, resulting in 10–70% less frozen precipitation (Fig. 6k, l). It is important to note that had the February 2010 AR had an even higher freezing level, impacts related to natural hazards could have been worse. Trends identified in this study suggest a greater likelihood of higher freezing level events in the futures, increasing the fraction of liquid to frozen precipitation, and subsequently increasing the risk for floods and landslides.

At the location of two of the landslides during the February 2010 AR, both events had similar IVT, but the January 1989 AR had higher magnitude water vapor flux (Fig. 9). Figure 11 shows the vertical profile of water vapor flux at two locations (Fig. 1a, black triangles) along western HMA range during both the 1989 and 2010 AR events and compares them to the water vapor flux during all Western HMA AR events. Both events fall above the 75th percentile, but the vertical distribution of the water vapor flux varies between the cases. At 34.87^∘^N, 72.66^∘^E (elevation is 1.96 km), water vapor flux during the January 1989 AR is strongest below 600 hPa, peaking at 0.085 m s^-1^ near 750 hPa. During the February 2010 AR, the distribution of water vapor flux was shifted to higher elevations, peaking at 0.07 m s^-1^ near 700 hPa, and dropping off more slowly above that (Fig. 11a). Results are similar for 34.09^∘^N and 74.02^∘^E, except the moisture flux for the February 2010 AR extended almost all the way to 400 hPa, peaking at 0.8 m s^-1^ between 750 and 600 hPa (Fig. 11b). Possible explanations for the deeper profiles of water vapor flux during the 2010 event include a stronger AR, a longer-duration AR (possibly allowing more time for moist parcels to rise), and a warmer air mass requiring more moisture to reach saturation. However, future work is needed to more fully quantify the relationships between AR / IVT intensity, duration, temperature, and the vertical profile of water vapor flux at inland locations.

## Conclusions

This study shows that between 1979 and 2015, southerly IVT has significantly increased in western India and Pakistan during Western HMA ARs, indicating that in recent decades, there has been an increase in the intensity of Western HMA ARs. Additionally, the height of the freezing level has significantly increased across southern Asia during HMA ARs. One consequence of these findings is that there is significantly less frozen precipitation during HMA ARs with an above-average freezing level compared to those with a below-average freezing level. Should future trends continue as currently observed, western HMA will see an increase in the intensity of ARs with an above-average freezing level. With more liquid precipitation during these events, there is a higher likelihood of risk for associated natural hazards such as landslides and floods.

To further highlight the importance of the freezing level on resulting precipitation in western HMA, this study focused on two impactful western HMA ARs: one that occurred during below-average freezing level conditions and one that occurred during above-average freezing level conditions. Both ARs transitioned from Northwestern to Western HMA ARs, were quasi-stationary over this area, featured greater than the 85th percentile of IVT for Western HMA ARs, and resulted in greater than the 85th percentile of precipitation for these storm types, largely due to a long duration of orographically lifted moisture within the AR plume. We used dynamically downscaled CFSR at 6.7 km spatial resolution to compare their mesoscale characteristics to determine the influence of the freezing level on orographic precipitation.

The below-average freezing level AR occurred in January 1989, lasted for just under 4 days, and resulted in about 175 mm of precipitation across western HMA. The above-average freezing level AR occurred in February 2010, lasted for about 5 days, resulted in about 200–450 mm of precipitation, and was related to six separate landslide events in western HMA. Although freezing levels were only 50–600 m higher during the 2010 AR, this event resulted in 10–70% less frozen precipitation than the 1989 AR (Fig. 6). This is an extreme difference between two disparate events, but even in aggregate from 1979 to 2015, there was a 10–40% reduction in frozen precipitation during above-average freezing level ARs (Fig. 4).

This study illustrates the importance of mesoscale conditions in modulating the interaction of ARs, topography, freezing level, and precipitation-triggered landslides. During the 2010 AR, a deep moist layer was orographically lifted directly perpendicular to the topography near the foothills of HMA, resulting in a combination of rain and snow of about 150 mm day^-1^. This triggered multiple landslides across western HMA near and upstream of where the freezing level intersected with the topography, in the transition zone from rain to snow. Future studies seeking to improve the predictive skill of these destructive events will therefore need to consider both the synoptic and mesoscale environments in which they occur.

While freezing level likely plays a large role in determining the likelihood of landslides, other factors are also important. Naturally, storm intensity and total precipitation (liquid or frozen) plays a role. Moderate, long-duration precipitation interspersed with short-duration high intensity precipitation increases the likelihood of precipitation-triggered shallow landslides (Cordeira et al. 2019; Kirschbaum et al. 2020; Oakley et al. 2018). Other factors that may need to be considered are antecedent soil moisture conditions, and the possibility of rain-on-snow events, which have been shown to increase the risk for floods and landslides when they occur (e.g., Guan et al. 2016).

In summary, this work conclusively shows that from 1979–2015 across HMA, the freezing level has increased (1–4%), the intensity of Western HMA ARs has increased (2–16% increase in IVT), and that when the freezing level is above-average, there is significantly less frozen precipitation. Furthermore, the examples of below- and above-average freezing level ARs presented here demonstrate the importance of mesoscale processes in orographic precipitation and highlight the varying outcomes that can result across HMA from relatively small differences in freezing level height.

## Data Availability

The AR data were provided by Bin Guan via https://dataverse.ucla.edu/dataverse/ar. Development of the AR detection algorithm and databases was supported by NASA. ERA5 data on single levels (Hersbach et al. 2018b, 10.24381/cds.adbb2d47) and pressure levels (Hersbach et al. 2018a, 10.24381/cds.bd0915c6) were downloaded from the Copernicus Climate Change Service (C3S) Climate Data Store. The results contain modified Copernicus Climate Change Service information 2020. Neither the European Commission nor ECMWF is responsible for any use that may be made of the Copernicus information or data it contains. The Global Landslide Catalog from (Kirschbaum et al. 2010, 2015) can be found at https://data.nasa.gov/Earth-Science/Global-Landslide-Catalog-Export/dd9e-wu2v. Climate Forecast System Reanalysis (CFSR) (Saha et al. 2010, https://rda.ucar.edu/datasets/ds093.0/) were dynamically downscaled using the Advanced Weather Research and Forecasting (ARW-WRF) modeling system version 3.7.1 (Skamarock et al. 2008, https://www2.mmm.ucar.edu/wrf/users/download/get_source.html).

## Appendix A: Calculation of IVT for WRF

Integrated water vapor transport (IVT), a variable widely used for the detection and identification of ARs (e.g., Guan and Waliser 2015; Ralph et al. 2019) is calculated by taking the 3-hourly model data, interpolating u and v wind components (m s^-1^), and water vapor mixing ratio (kg kg^-1^) to 20 pressure levels (1000, 975, 950, 925, 900, 875, 850, 825, 800, 775, 750, 700, 650, 600, 550, 500, 450, 400, 350, and 300 hPa). Only data at pressure levels above ground level were used for each grid cell in the integration. Then, using water vapor mixing ratio, we computed specific humidity (q) and then integrated u and v wind components with q at all pressure levels above ground level using the following equations:

1 $$\begin{aligned} IVT_{x}= & {} -\frac{1}{g} \int _{1000}^{300} u q dp \end{aligned}$$

2 $$\begin{aligned} IVT_{y}= & {} -\frac{1}{g} \int _{1000}^{300} v q dp \end{aligned}$$

where *g* is the gravitational acceleration (m $\hbox {s}^{-2}$), *u* is zonal wind (m s^-1^), *v* is meridional wind (m s^-1^), *q* is specific humidity (kg kg^-1^), *p* is pressure (Pa = kg m^-1^ $\hbox {s}^{-2}$), and the column integration is between pressure levels 1000 and 250 hPa inclusive.

The magnitude of IVT is calculated using the following equation:

3 $$\begin{aligned} IVT = \sqrt{IVT_{x}^2 + IVT_{y}^2} \end{aligned}$$

Specific humidity (kg kg ^-1^) is derived from water vapor mixing ratio (kg kg ^-1^) using the formula from Wallace and Hobbs (2006) where q is specific humidity and w is the water vapor mixing ratio.

4 $$\begin{aligned} q = \frac{w}{1 + w} \end{aligned}$$

## Appendix B: Calculation of water vapor flux

Water vapor flux, a variable used in multiple AR-related studies to examine the vertical profile of water vapor (e.g., Guan and Waliser 2015), is the flux of water vapor at each identified pressure level. The following equations were used to calculate water vapor flux:

5 $$\begin{aligned}{} & {} VT_{u}^{p} = q*u \end{aligned}$$

6 $$\begin{aligned}{} & {} VT_{v}^{p} = q*v \end{aligned}$$

7 $$\begin{aligned}{} & {} VT = \sqrt{VT^{2}_{u} + VT^{2}_{v}} \end{aligned}$$

where *q* is specific humidity (kg kg^-1^), *u* is zonal wind (m s^-1^), v is meridional wind (m s^-1^) at specified pressure, *p*. If specific humidity retains its kg kg^-1^, the resulting units for water vapor flux are m s^-1^.

## Code availability

The code for this analysis can be found at https://github.com/dlnash/HMA_AR_freezing_level.

## References

[CR1] Cannon F, Hecht CW, Cordeira JM, Ralph FM (2018). Synoptic and mesoscale forcing of Southern California extreme precipitation. J Geophys Res Atmos.

[CR2] Cordeira JM, Stock J, Dettinger MD, Young AM, Kalansky JF, Ralph FM (2019). A 142-year climatology of northern California landslides and atmospheric rivers. Bull Am Meteorol Soc.

[CR3] Espinoza V, Waliser DE, Guan B, Lavers DA, Ralph FM (2018). Global analysis of climate change projection effects on atmospheric rivers. Geophys Res Lett.

[CR4] Guan B, Waliser DE (2015). Detection of atmospheric rivers: evaluation and application of an algorithm for global studies. J Geophys Res Atmos.

[CR5] Guan B, Waliser DE (2019). Tracking atmospheric rivers globally: spatial distributions and temporal evolution of life cycle characteristics. J Geophys Res Atmos.

[CR6] Guan B, Waliser DE, Ralph FM, Fetzer EJ, Neiman PJ (2016). Hydrometeorological characteristics of rain-on-snow events associated with atmospheric rivers. Geophys Res Lett.

[CR7] Harris J, Bowman KP, Shin DB (2000). Comparison of freezing-level altitudes from the NCEP reanalysis with TRMM precipitation radar brightband data. J Clim.

[CR8] Hersbach H, Bell B, Berrisford P, Biavati G, Horányi A, Muñoz Sabater J, Nicolas J, Peubey C, Radu R, Rozum I, Schepers D, Simmons A, Soci C, Dee DP, Thépaut JN (2018a) ERA5 hourly data on pressure levels from 1979 to present. 10.24381/cds.bd0915c6

[CR9] Hersbach H, Bell B, Berrisford P, Biavati G, Horányi A, Muñoz Sabater J, Nicolas J, Peubey C, Radu R, Rozum I, Schepers D, Simmons A, Soci C, Dee DP, Thépaut JN (2018b) ERA5 hourly data on single levels from 1979 to present. 10.24381/cds.adbb2d47

[CR10] Hersbach H, Bell B, Berrisford P, Hirahara S, Horányi A, Muñoz-Sabater J, Nicolas J, Peubey C, Radu R, Schepers D, Simmons A, Soci C, Abdalla S, Abellan X, Balsamo G, Bechtold P, Biavati G, Bidlot J, Bonavita M, De Chiara G, Dahlgren P, Dee DP, Diamantakis M, Dragani R, Flemming J, Forbes R, Fuentes M, Geer A, Haimberger L, Healy S, Hogan RJ, Hólm E, Janisková M, Keeley S, Laloyaux P, Lopez P, Lupu C, Radnoti G, de Rosnay P, Rozum I, Vamborg F, Villaume S, Thépaut JN (2020). The ERA5 global reanalysis. Q J R Meteorol Soc.

[CR11] Hewitt K (2005). The Karakoram Anomaly? Glacier Expansion and the ‘Elevation Effect’. Karakoram Himalaya. Mt Res Dev.

[CR12] Hong SY, Noh Y, Dudhia J (2006). A new vertical diffusion package with an explicit treatment of entrainment processes. Mon Weather Rev.

[CR13] Iacono MJ, Delamere JS, Mlawer EJ, Shephard MW, Clough SA, Collins WD (2008). Radiative forcing by long-lived greenhouse gases: calculations with the AER radiative transfer models. J Geophys Res Atmos.

[CR14] Kääb A, Berthier E, Nuth C, Gardelle J, Arnaud Y (2012). Contrasting patterns of early twenty-first-century glacier mass change in the Himalayas. Nature.

[CR15] Kain JS (2004). The Kain-Fritsch convective parameterization: an update. J Appl Meteorol Climatol.

[CR16] Kirschbaum D, Adler R, Hong Y, Hill S, Lerner-Lam A (2010). A global landslide catalog for hazard applications: method, results, and limitations. Nat Hazards.

[CR17] Kirschbaum D, Stanley T, Zhou Y (2015). Spatial and temporal analysis of a global landslide catalog. Geomorphology.

[CR18] Kirschbaum D, Kapnick SB, Stanley T, Pascale S (2020). Changes in extreme precipitation and landslides over high mountain Asia. Geophys Res Lett.

[CR19] Lang TJ, Barros AP (2004). Winter storms in the central Himalayas. J Meteorol Soc Japan.

[CR20] Lundquist JD, Neiman PJ, Martner B, White AB, Gottas DJ, Ralph FM (2008). Rain versus snow in the Sierra Nevada, California: comparing doppler profiling radar and surface observations of melting level. J Hydrometeorol.

[CR21] Minder JR, Durran DR, Roe GH (2011). Mesoscale controls on the mountainside snow line. J Atmos Sci.

[CR22] Monin AS, Obukhov AM (1954). Basic laws of turbulent mixing in the surface layer of the atmosphere. Tr Akad Nauk SSSR Geophiz Inst.

[CR24] Nash D, Carvalho LMV (2020). Brief Communication: An electrifying atmospheric river - understanding the thunderstorm event in Santa Barbara County during March 2019. Nat Hazards Earth Syst Sci.

[CR25] Nash D, Carvalho LMV, Jones C, Ding Q (2021). Winter and spring atmospheric rivers in High Mountain Asia: climatology, dynamics, and variability. Clim Dyn.

[CR26] Neiman PJ, Ralph FM, Wick GA, Lundquist JD, Dettinger MD (2008). Meteorological characteristics and overland precipitation impacts of atmospheric rivers affecting the west coast of North America based on eight years of SSM/I satellite observations. J Hydrometeorol.

[CR27] Neiman PJ, Schick LJ, Ralph FM, Hughes M, Wick GA (2011). Flooding in Western Washington: the connection to atmospheric rivers. J Hydrometeorol.

[CR28] Niu GY, Yang ZL, Mitchell KE, Chen F, Ek MB, Barlage M, Kumar A, Manning K, Niyogi D, Rosero E, Tewari M, Xia Y (2011). The community Noah land surface model with multiparameterization options (Noah-MP): 1 Model description and evaluation with local-scale measurements. J Geophys Res Atmos.

[CR29] Norris J, Carvalho LMV, Jones C, Cannon F (2015). WRF simulations of two extreme snowfall events associated with contrasting extratropical cyclones over the western and central Himalaya. J Geophys Res Atmos.

[CR30] Norris J, Carvalho LMV, Jones C, Cannon F, Bookhagen B, Palazzi E, Tahir AA (2017). The spatiotemporal variability of precipitation over the Himalaya: evaluation of one-year WRF model simulation. Clim Dyn.

[CR31] Norris J, Carvalho LMV, Jones C, Cannon F (2019). Deciphering the contrasting climatic trends between the central Himalaya and Karakorum with 36 years of WRF simulations. Clim Dyn.

[CR32] Oakley NS, Lancaster JT, Kaplan ML, Ralph FM (2017). Synoptic conditions associated with cool season post-fire debris flows in the Transverse Ranges of southern California. Nat Hazards.

[CR33] Oakley NS, Lancaster JT, Hatchett BJ, Stock J, Ralph FM, Roj S, Lukashov S (2018). A 22-year climatology of cool season hourly precipitation thresholds conducive to shallow landslides in California. Earth Interact.

[CR34] Ralph FM, Rutz JJ, Cordeira JM, Dettinger MD, Anderson M, Reynolds D, Schick LJ, Smallcomb C (2019). A scale to characterize the strength and impacts of atmospheric rivers. Bull Am Meteorol Soc.

[CR35] Rutz JJ, Steenburgh WJ, Ralph FM (2014). Climatological characteristics of atmospheric rivers and their inland penetration over the Western United States. Mon Weather Rev.

[CR36] Saha S, Moorthi S, Pan HL, Wu X, Wang JJ, Nadiga S, Tripp P, Kistler R, Woollen J, Behringer D, Liu H, Stokes D, Grumbine R, Gayno G, Wang JJ, Yt Hou, Hy Chuang, Juang HMH, Sela J, Iredell M, Treadon R, Kleist D, Delst PV, Keyser D, Derber J, Ek MB, Meng J, Wei H, Yang R, Lord S, Hvd Dool, Kumar A, Wang W, Long C, Chelliah M, Xue Y, Huang B, Jk Schemm, Ebisuzaki W, Lin R, Xie P, Chen M, Zhou S, Higgins RW, Cz Zou, Liu Q, Chen Y, Han Y, Cucurull L, Reynolds RW, Rutledge G, Goldberg M (2010). The NCEP climate forecast system reanalysis. Bull Am Meteorol Soc.

[CR37] Skamarock WC, Klemp JB, Dudhia J, Gill DO, Barker DM, Wang W, Powers JG (2008) A description of the advanced research WRF Version 3. Tech. rep., National Center for Atmospheric Research, Boulder, CO, USA, 10.5065/D68S4MVH

[CR38] Stauffer DR, Seaman NL (1990). Use of four-dimensional data assimilation in a limited-area mesoscale model. Part I: Experiments with synoptic-scale data. Mon Weather Rev.

[CR39] Stauffer DR, Seaman NL, Binkowski FS (1991). Use of four-dimensional data assimilation in a limited-area mesoscale model Part II: Effects of data assimilation within the planetary boundary layer. Mon Weather Rev.

[CR40] Thapa K, Endreny TA, Ferguson CR (2018). Atmospheric rivers carry nonmonsoon extreme precipitation into Nepal. J Geophys Res Atmos.

[CR41] Thompson G, Field PR, Rasmussen RM, Hall WD (2008). Explicit forecasts of winter precipitation using an improved bulk microphysics scheme. Part II: Implementation of a new snow parameterization.. Mon Weather Rev.

[CR42] Wallace JM, Hobbs PV (2006). Atmospheric science: an introductory survey.

[CR43] Wang S, Zhang M, Pepin NC, Li Z, Sun M, Huang X, Wang Q (2014). Recent changes in freezing level heights in High Asia and their impact on glacier changes. J Geophys Res Atmos.

[CR44] Yang Y, Zhao T, Ni G, T Sun (2018). Atmospheric rivers over the Bay of Bengal lead to northern Indian extreme rainfall. Int J Climatol.

[CR45] Zhu Y, Newell RE (1994). Atmospheric rivers and bombs. Geophys Res Lett.

